# The Migration Pattern of a Short-Tapered Femoral Stem Correlates with the Occurrence of Cortical Hypertrophies: A 10-Year Longitudinal Study Using Ein Bild Röntgen Analyse–Femoral Component Analysis

**DOI:** 10.3390/jcm13123616

**Published:** 2024-06-20

**Authors:** Tobias Freitag, Michael Fuchs, David Friedrich, Ralf Bieger, Heiko Reichel, Moritz Oltmanns

**Affiliations:** 1 Department of Orthopaedic Surgery, University of Ulm, Oberer Eselsberg 45, 89081 Ulm, Germany; michael.fuchs@rku.de (M.F.); david.friedrich@uniklinik-ulm.de (D.F.); sekretariat.orthopaedie@rku.de (H.R.); moritz.oltmanns@rku.de (M.O.); 2 Center for Knee, Hip and Shoulder Surgery, Schoen Clinic München Harlaching, Harlachinger Strasse 51, 81547 Munich, Germany; ralf.bieger@uni-ulm.de

**Keywords:** total hip arthroplasty, short stem, patient-reported outcome measures, stem migration, survival analysis

## Abstract

**Background:** Shorter hip stems have shown promising mid-term results but lack long-term data. High rates of distal cortical hypertrophy (CH) have been described, suggesting a more diaphyseal load transmission. This study aimed to determine patient-specific and surgery-related factors influencing CH and their impact on 10-year outcomes. **Methods**: It included 100 consecutive total hip arthroplasties (THAs) using the Fitmore stem (Zimmer, Warsaw, Indiana), with clinical and radiographic follow-ups at 1, 2, 5, and at least 10 years post-surgery. **Results**: No revisions were performed due to aseptic loosening after a mean of 11.6 years (range: 10–13.5 years). CH was observed in 26% of hips, primarily in Gruen zones 3 and 5. There was no significant difference in the Harris Hip Score between patients with and without CH. Larger stem sizes and greater axial subsidence significantly correlated with CH occurrence (OD 1.80, (1.13–1.92), *p* = 0.004; OD 1.47, (1.04–2.08), *p* = 0.028). The Fitmore stem demonstrated excellent survival rates and favorable outcomes over 10 years. **Conclusions**: Despite a lower CH rate compared to other studies, significant correlations with stem size and subsidence were identified. This study underscores the importance of patient selection and achieving high primary stability to maintain the metaphyseal anchoring concept.

## 1. Introduction

Given the high success rates of modern total joint arthroplasty (THA), the volume of primary THA has continuously increased in recent decades [1,2]. Moreover, a further substantial increase is predicted for many countries in the coming years [3,4]. As part of this trend, the use of shorter femoral stem designs, which enable minimally invasive and more bone-preserving implantation, is likewise increasing [5,6]. The Fitmore stem (Zimmer Biomet, Warsaw, IN, USA) was introduced in 2007 and is, meanwhile, a commonly used stem model in elective THA in Germany, with more than 27,000 implantations recorded in 2022 [5]. Encouraging mid-term results have been reported [7], leading to a 10A* rating by the Orthopedic Data Evaluation Panel (ODEP). However, while established standard stems have demonstrated excellent long-term survival with good clinical and radiological outcomes over up to 25 years [8], data on long-term follow-up studies of the Fitmore stem are lacking. Although initial studies show positive results after 10 years [9], long-term survivorship still requires investigation. With its proximal coating, the Fitmore stem aims to facilitate proximal load transfer, reducing stress shielding and providing a more physiological bone strain. This concept has been partially supported in biomechanical and clinical studies [10,11]. Conversely, radiological findings, especially cortical hypertrophy (CH) in Gruen zones 3 and 5, suggest a more distal load transfer and increased proximal stress shielding than estimated [9,12]. CH has been reported in longitudinal studies of the Fitmore stem, with rates ranging between 20–70% after two to five years [13,14]. The relevance of CH remains not fully understood. Cortical hypertrophy is a known phenomenon, lacking a consistent definition, and has been observed in nearly all types of uncemented stems [15]. Initially suspected to cause thigh pain specific to short stems [16], various studies have shown no correlation between cortical hypertrophy and these symptoms [17,18]. Several factors have been suggested to contribute to cortical hypertrophy. Increased head diameter, leading to more friction and increased load transmission on the stem tip, has been identified as a risk factor [19]. Additionally, large implant dimensions appear to correlate with the occurrence of CH [14]. Other authors have described an enlarged femoral offset as correlating with higher rates of CH in cementless THA [12]. Moreover, the canal fill index (CFI) and cortical thickness index (CI) were discussed in this context [20]. However, the presence of cortical hypertrophy does not seem to affect patient-reported outcome measures (PROMs), and so far, no correlation with aseptic loosening has been found within the first five years, suggesting that cortical hypertrophy may not be a radiological finding of significant concern [21].

Stem subsidence has been identified as a predictor for long-term survivorship of cementless femoral implants [22]. For cementless stems, initial subsidence is common, with implant stabilization typically occurring within the first 48 months [23,24,25,26]. For the Fitmore stem, mean subsidence ranging from 1.1 to 1.9 mm in the first five years post-surgery has been described, mostly without further subsidence after two years, and with a reported survival rate of 99% after 5 years [21,27]. A threshold has not been defined yet since higher mean subsidence in the mid-term was reported without stem-related revision within 10 years [9]. For their collective, the authors reported a CH rate of 74% after 5 years. However, there is considerable paucity in the literature with regard to long-term results beyond 5–10 years [9]. This study represents the continuation of prospective observation of our first 100 Fitmore-stem implantations. The main focus was to investigate the relationship between patient-specific parameters, stem subsidence, and the occurrence of CH with a minimum follow-up of 10 years.

## 2. Materials and Methods

### 2.1. Study Design

The present retrospective diagnostic cohort study examined our first 100 THAs using the Fitmore stem between 2008 and 2009. The study received approval from the local ethical review board (Approval No: 365/12) and was conducted in accordance with the principles outlined in the Helsinki Declaration of 2008. The senior authors have previously published the short- and mid-term results of this study group, reporting initial stem subsidence with secondary stabilization after two years [24,27].

Inclusion criteria were written consent for participation as well as at least four radiographs with a minimum follow-up of 10 years meeting the requirements for examination with EBRA-FCA (Ein Bild Röntgen Analyse–Femoral Component Analysis), as detailed in our previous studies [24,27].

### 2.2. Patients and Demographics

At the final follow-up, data from 75 patients (77 hips) were available. Seven patients died, one patient underwent stem revision due to traumatic periprosthetic femoral fracture, one patient underwent revision due to periprosthetic joint infection 1 year after surgery followed by a two-stage revision, one patient rejected further participation, and thirteen were lost to follow-up. Thirty-seven hips (48%) were female. The mean age at the time of primary THA was 67 years (range: 36–86 years), and the mean follow-up was 11.6 years (range: 10–13.5 years).

### 2.3. Surgeries and Implant Characteristics

Surgery was performed by five experienced senior orthopedic surgeons using an anterolateral or a modified lateral approach in the supine position. All patients received a cementless press-fit acetabular cup (Allofit *n* = 52, Trilogy *n* = 25; Zimmer, Warsaw, IN, USA) and an alumina-on-highly crosslinked polyethylene bearing with a 32 mm head diameter.

The rehabilitation protocol included full weight-bearing using two crutches immediately after surgery. Radiological and clinical follow-ups were scheduled immediately after surgery, 3 and 12 months, 2 and 5 years, and after a minimum of 10 years. In addition to a clinical examination and questioning, X-rays (pelvis a.p. and Sven Johansson view of the affected hip) were taken at each outpatient presentation.

**Figure 1:**
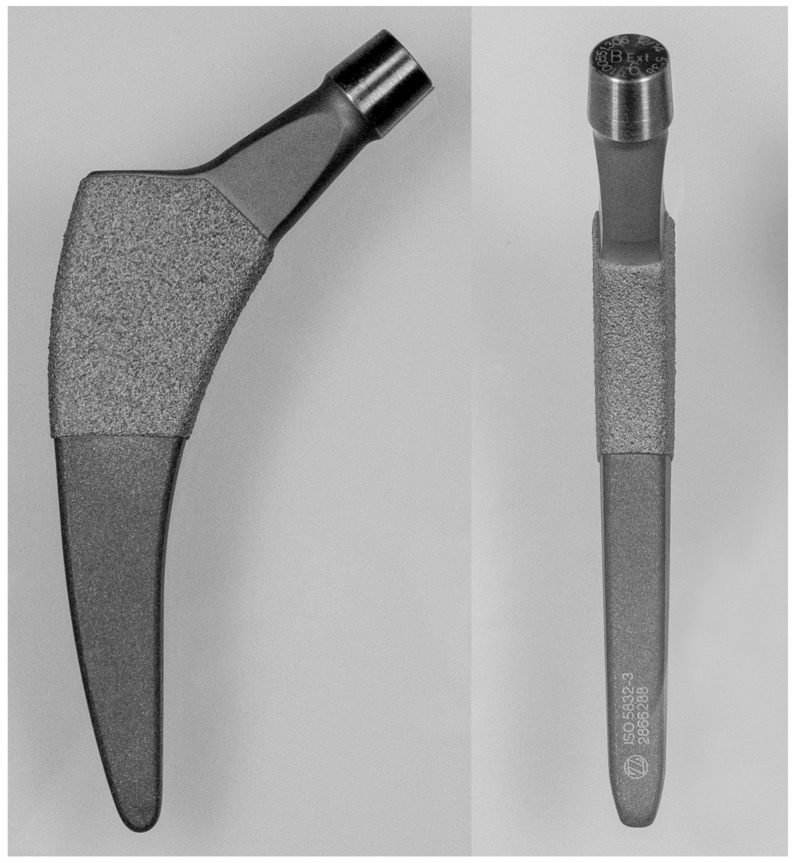
Photograph in two planes of Fitmore hip stem.

The Fitmore stem is a trochanter-sparing femoral short stem made of titanium alloy (Ti Al6V4). Its proximal portion features a plasma-coated surface to facilitate a metaphyseal press-fit and promote bony ingrowth (Figure 1).

This stem aligns with the type 4 classification outlined by Khanuja et al. [28], characterized by a shortened conventional design with primary fixation in the proximal metaphysis. The collarless stem boasts a tapered profile in three planes, presenting a trapezoidal cross-section. It is offered in three stem families (A, B, B extended, and C), each with varying degrees of medial curvature. The radius of the medial curvature decreases from family A to C, with the goal of restoring the individual’s anatomical alignment.

### 2.4. Clinical and Radiographic Evaluation

Radiographs were evaluated by one reviewer who was not involved in index surgery for radiolucent lines, osteolysis, heterotopic ossifications, implant loosening, and cortical hypertrophies [29,30]. Axial stem migration was measured using EBRA-FCA. For this purpose, 19 reference points in all a.p. radiographs were determined, which are necessary for the assessment of comparability of the X-ray images and the measurements as previously described [24,27]. Changes in hip offset, cortical index, and canal fill index were measured using an orthopedic planning tool (mediCAD^®^, Hectec, Altdorf/Landshut, Germany); calibration was performed using the head diameter as well. The cortical index (CI) was measured 10 cm below the apex of the lesser trochanter [31]. The canal fill index (CFI) was calculated using the mean of three measurements 2 cm above, at, and 2 cm below the lesser trochanter [32]. Change in hip offset was measured between the teardrop figure and the femoral shaft axis [33] and was presented as a percentage of change. Cortical hypertrophy was defined as any thickening of the external cortex located along the stem-adapted Gruen zones [30]. Preoperatively and at each time of follow-up, the Harris hip score (HHS) was assessed.

### 2.5. Statistics

Results were reported as the number of observations with percentages for categorical data, and comparisons were conducted using the chi-squared test. Data values are expressed as means with ranges; comparisons were performed using independent-sample *t*-tests. Logistic regression analysis was employed to analyze the risk factors for developing cortical hypertrophy (CH). Kaplan–Meier survival analysis was performed with all stem revisions as the endpoint. All statistical analyses were conducted using SPSS Version 29.0 (IBM SPSS Statistics, IBM, Armonk, NY, USA). A significance level of *p* < 0.05 was considered statistically significant.

## 3. Results

After a minimum follow-up of 10 years, no revisions were performed due to aseptic loosening. The mean Harris hip score changed from 58 preoperatively to 89 (range: 42–98) at the two-year follow-up. After 5 and 10 years, a consistent HHS of 90 and 89 was observed (see Table 1).

**Figure 2:**
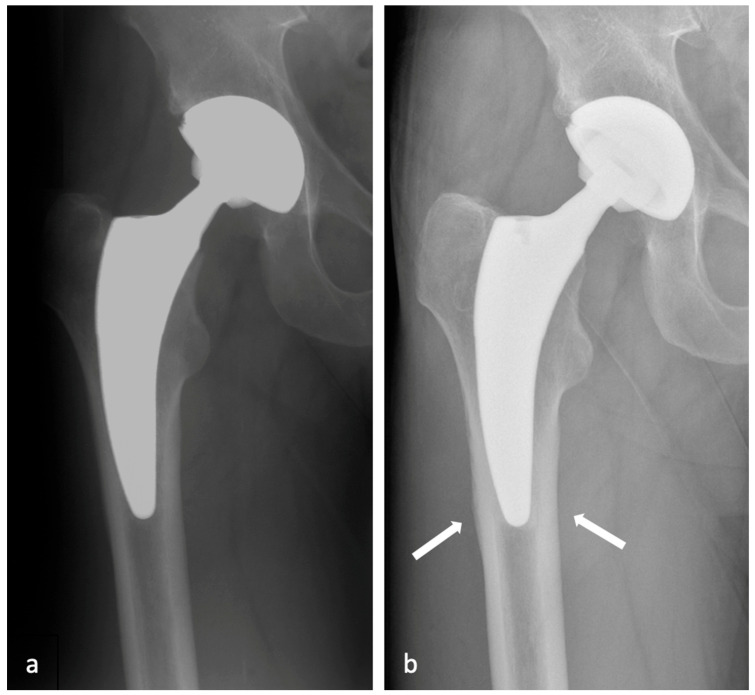
(**a**,**b**) X-rays taken 5 days postoperatively (**a**) in a 57-year-old male and after 11 years (**b**) of a representative case of cortical hypertrophy typically in zones 3 and 5 according to Gruen.

In the radiological assessment, no radiolucent lines were observed at the implant–bone interface of the femur in any case. Cortical hypertrophy (CH) was present in 20 hips (26%) and remained largely stable after 2 years, exclusively located in Gruen zones 3 and 5 (see Figure 2).

**Figure 3:**
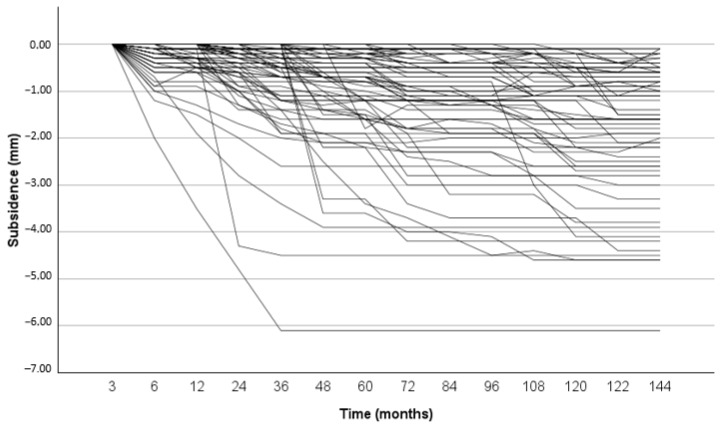
Graph showing individual axial stem migration over time (*n* = 77).

**Figure 4:**
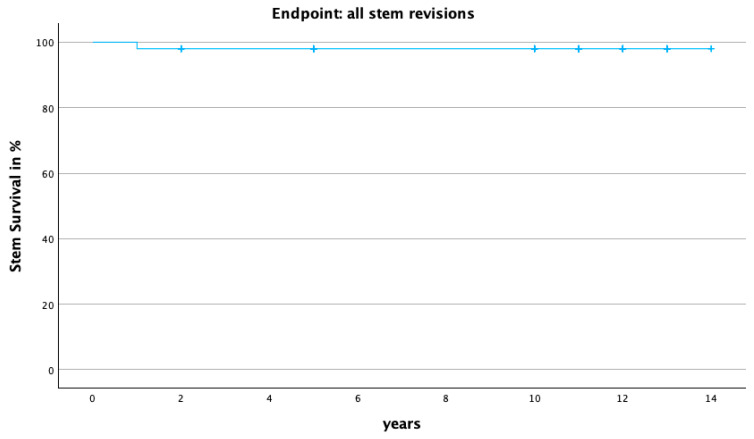
Kaplan–Meier survival curve for endpoint “all stem revisions” (98%; 95%-CI; 72.3–99.6%; *n* = 100).

Axial stem subsidence was initially observed up to 2 years, with 18 implants exhibiting early-onset subsidence > 1.5 mm, followed by a stable implant position after a minimum of 10 years (see Figure 3). The mean subsidence was 1.1 mm (−5.0 mm to 1.5 mm) after 5 years and 1.4 mm (−6.1 mm to 0.8 mm) after a minimum of 10 years. There was no significant difference in age, gender, BMI, diagnosis, and HHS between patients with and without CHs (see Table 1). The Kaplan–Meier survival rate after 10 years for all stem revisions as the endpoint was 98% (95% CI: 72.3–99.6%; Figure 4), with no association between CH and stem revision. There were no stem revisions due to aseptic stem loosening.

**Figure 5:**
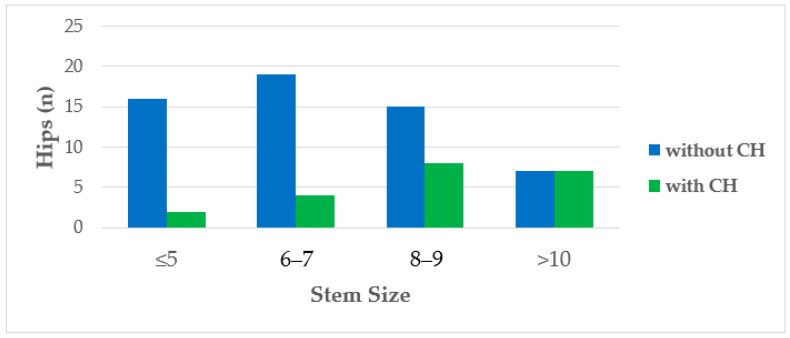
Histogram depicting the distribution and ratio of hips with and without cortical hypertrophy (CH) based on the size of the femoral implant used. The utilization of larger implant dimensions was correlated with a higher rate of CHs (*n* = 77).

**Figure 6:**
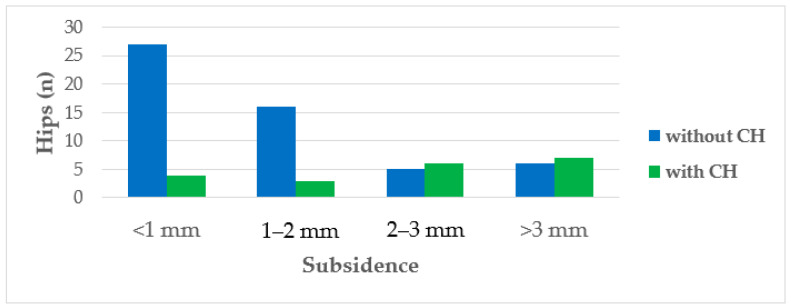
Histogram illustrating the distribution and ratio of hips with and without cortical hypertrophy (CH) based on the amount of axial stem migration. The subcategories of 2–3 mm and >3 mm subsidence exhibited a higher proportion of hips with CHs (*n* = 77).

CFI (0.79; 0.54–0.95), CI (0.57; 0.36–0.83), and change in hip offset (1.3%; −21.3–23.8%) did not differ significantly in the prevalence of CH (Table 2). Two variables demonstrated a significant correlation with CH in a logarithmic regression model. Larger stem size was associated with a higher likelihood of developing CH (*p* = 0.004, 95% CI 1.13–1.29, OR 1.80, Figure 5), as well as greater axial stem subsidence (*p* = 0.028, 95% CI 1.04–2.08, OR 1.47, Figure 6).

## 4. Discussion

The design concept of the Fitmore stem includes a metaphyseal load transmission to mitigate stress shielding effects, as observed in conventional implant designs. A more physiological load transmission compared to a standard straight stem was confirmed in a randomized dual-energy X-ray absorptiometry study over a period of 1 year [11]. Nevertheless, recent reports indicate high rates of cortical hypertrophy between 56% and 74% for this stem [9,12], suggesting a more diaphyseal load transmission in these cases, which contradicts the intended concept. This observation is significant because stress shielding effects have been linked to aseptic loosening [34], which may be particularly relevant for shorter implant designs. In addition to monitoring implant survival, it is crucial for clinical applications to identify risk factors to facilitate consistent implementation of the metaphysical anchoring concept.

In this study, an excellent overall survival rate of 98% was observed for the Fitmore stem after a 10-year follow-up period, with no revisions due to aseptic stem loosening. These results are comparable to well-established cementless standard stems, such as the CLS Spotorno stem (Zimmer, Warsaw, IN, USA), which maintain survival rates well over 90% even at 20 years [35]. Long-term studies typically emphasize implant stability and survival rather than patient-related outcome scores [36]. However, it is worth noting that patient-related outcome measures remain consistently excellent after 10 years and were not adversely affected by the occurrence of CH.

We observed a significantly lower rate of CH at 26% compared to the literature. Schader et al. reported a prevalence of 74% (59 hips) with a mean stem subsidence of 5 mm over 10 years, which is in contrast to our findings of 1.4 mm after the same period. The higher migration pattern in their cohort of 80 hips may explain the significantly higher prevalence of CH, consistent with our results, which demonstrated a significant correlation between stem subsidence and CH prevalence. This point should be underscored, as the authors reported a mean stem subsidence of 1.93 mm in this cohort after 5 years [21], with the majority of migration typically occurring within the first two years [25,26,27]. However, migration measurements were conducted using only two reference points without considering the comparability of radiographs, resulting in lower data reliability [37]. The most reliable method for measuring stem subsidence on standard anteroposterior radiographs without additional means at exposure is EBRA-FCA, with an accuracy of 1 mm [38]. Our study found a mean subsidence of 1.4 mm after 10 to 14.5 years, with 18 hips showing subsidence > 1.5 mm within the first two years, and a significant correlation with CH occurrence. Our interpretation of this observation includes distal cortical implant contact resulting from relevant subsidence, leading to loss of primary proximal fixation by the overcoated proximal implant third. To our knowledge, this is the first study to highlight the association between CH prevalence and stem subsidence using a highly reliable method.

A similarly high rate of cortical hypertrophies (CHs) at 54% after a mean of 7.7 years was reported by Innmann et al. [12]. In a cohort of 188 hips, they identified a significant correlation between the postoperative change in hip offset and the occurrence of CHs. Patients with adequate or over-reconstructed hip offset demonstrated a higher proportion of hips with cortical hypertrophies. However, due to the fact that adequate reconstruction of hip geometry is the desired goal, the authors concluded that CHs must be accepted to achieve this by the use of the evaluated short stem. In our study group, we did not observe any significant correlation with a change in hip offset, which may be attributed to the smaller cohort size. A higher, especially lateral, load transmission at the lower third of the implant seems plausible with an increase in offset, particularly given the short, curved stem design.

Nevertheless, our results revealed a correlation with large implant sizes. Stem rigidity is a crucial parameter for bone remodeling processes. Stems with greater flexibility have demonstrated the potential to reduce proximal bone loss and prevent cortical hypertrophies at the distal part of the stem [39]. In a biomechanical study investigating mediolateral implant-bending behavior, the Fitmore stem exhibited significantly higher rigidity compared to a cementless straight stem [40]. Stem rigidity increases with stem size, resulting in an enhanced load transfer in the distal region around the implant tip, which aligns with the observed cortical hypertrophies exclusively located in zones 3 and 5 according to Gruen [41].

Some limitations of our study have to be mentioned. First, this study relies on a relatively small sample size, primarily due to the retrospective study design, and patients lost to follow-up and death within the study period. Due to these aspects and the fact that no preliminary power analysis was carried out, risk calculations of possible influencing factors for the occurrence of cortical hypertrophies using logistic regression analysis may be underpowered and should be interpreted cautiously. Nonetheless, several cited studies up to 10 years reported similar or higher dropout rates [9,12]. Second, the study cohort comprises our initial 100 implantations with this short stem, potentially introducing a preoperative selection bias. Third, the measurement methods employed only permit an indirect assessment of bony remodeling processes, and anatomical parameters as well as implant position were solely evaluated in the frontal plane.

## 5. Conclusions

The Fitmore stem demonstrates excellent results over a 10-year follow-up period, boasting high survival rates and favorable clinical outcomes regardless of the occurrence of CH. We identified increased stem size and particularly stem subsidence as risk factors for developing CHs, underscoring the importance of patient selection and achieving high primary implant stability to successfully implement the metaphyseal anchoring concept. We have no concerns regarding the second decade with this shorter stem; however, we acknowledge the necessity for further monitoring of the potential impact of CHs on long-term survival.

## Figures and Tables

**Table 1:** Demographics and diagnosis for patients with and without cortical hypertrophies.

	without CH	with CH	*p*-Value
Demographics			
Number of hips	57	20	
Gender (m:f)	29:28	11:9	0.33
Age at surgery in years	56 (37–75)	52 (23–69)	0.71
BMI (kg/m^2^)	27 (21–32)	26 (19–30)	0.37
HHS preoperatively	58 (42–68)	60 (52–66)	0.58
HHS postoperatively (2 y FU)	89 (87–97)	91 (77–98)	0.38
HHS postoperatively (5 y FU)	90 (89–98)	90 (78–99)	0.67
HHS postoperatively (min. 10 y FU)	89 (88–98)	91 (76–98)	0.42
Diagnosis			
Primary osteoarthritis	28	9	0.69
Avascular necrosis	6	3	0.21
Developmental dysplasia	19	6	0.19
Perthes disease	1	0	0.11
Posttraumatic	1	1	0.26
Protrusio acetabuli	2	1	0.88

CH, cortical hypertrophy; FU, follow-up. Data are presented as mean and range; f, female; m, male; BMI, body mass index; HSS, Harris hip score.

**Table 2:** Logistic regression analysis of risk factors for developing cortical hypertrophy.

Model (*n* = 77)	Odds Ratio (95%-CI)	*p*-Value
CFI	3.11 (0.12–80.7)	0.64
CI	0.33 (0.03–4.28)	0.12
Stem size	1.80 (1.13–1.92)	0.004 *
∇ Hip offset	1.01 (0.96–1.07)	0.702
Stem Subsidence	1.47 (1.04–2.08)	0.028 *

CFI, canal fill index; CI, cortical index. * highlighting significance (*p* < 0.05).

## Data Availability

The original contributions presented in the study are included in the article, further inquiries can be directed to the corresponding author.
